# Down-Regulation of Complement Receptors on the Surface of Host Monocyte Even as *In Vitro* Complement Pathway Blocking Interferes in Dengue Infection

**DOI:** 10.1371/journal.pone.0102014

**Published:** 2014-07-25

**Authors:** Cintia Ferreira Marinho, Elzinandes Leal Azeredo, Amanda Torrentes-Carvalho, Alessandro Marins-Dos-Santos, Claire Fernandes Kubelka, Luiz José de Souza, Rivaldo Venâncio Cunha, Luzia Maria de-Oliveira-Pinto

**Affiliations:** 1 Laboratory of Viral Immunology, Instituto Oswaldo Cruz, Rio de Janeiro, Brazil; 2 Flow Cytometry Sector, Centre for Cell Sorting and Analysis, Instituto Oswaldo Cruz, Rio de Janeiro, Brazil; 3 Referral Centre for Dengue, Campos de Goytacazes, Brazil; 4 Department of Clinical Medicine, Universidade Federal do Mato Grosso do Sul, Campo Grande, Brazil; Duke-National University of Singapore Graduate Medical School, Singapore

## Abstract

In dengue virus (DENV) infection, complement system (CS) activation appears to have protective and pathogenic effects. In severe dengue fever (DF), the levels of DENV non-structural-1 protein and of the products of complement activation, including C3a, C5a and SC5b-9, are higher before vascular leakage occurs, supporting the hypothesis that complement activation contributes to unfavourable outcomes. The clinical manifestations of DF range from asymptomatic to severe and even fatal. Here, we aimed to characterise CS by their receptors or activation product, *in vivo* in DF patients and *in vitro* by DENV-2 stimulation on monocytes. In comparison with healthy controls, DF patients showed lower expression of CR3 (CD11b), CR4 (CD11c) and, CD59 on monocytes. The DF patients who were high producers of SC5b-9 were also those that showed more pronounced bleeding or vascular leakage. Those findings encouraged us to investigate the role of CS *in vitro*, using monocytes isolated from healthy subjects. Prior blocking with CR3 alone (CD11b) or CR3 (CD11b/CD18) reduced viral infection, as quantified by the levels of intracellular viral antigen expression and soluble DENV non-structural viral protein. However, we found that CR3 alone (CD11b) or CR3 (CD11b/CD18) blocking did not influence major histocompatibility complex presentation neither active caspase-1 on monocytes, thus probably ruling out inflammasome-related mechanisms. Although it did impair the secretion of tumour necrosis factor alpha and interferon alpha. Our data provide strategies of blocking CR3 (CD11b) pathways could have implications for the treatment of viral infection by antiviral-related mechanisms.

## Introduction

By augmenting the neutralising activity of antiviral antibodies, complement activation inhibits infection with many viruses [1], [2], [3], [4], [5]. In flavivirus infection, such activation appears to play a protective or a pathogenic role, depending on the immune status of the host, the specific virus and infectious phase [6], [7]. The dengue virus (DENV), a member of the *Flavivirus* genus, comprises four closely related and antigenically distinct serotypes (DENV-1 through DENV-4). The flavivirus virion consists of a nucleocapsid surrounded by a lipid bilayer containing three structural proteins (the envelope, capsid and membrane proteins) and seven non-structural proteins (NS1, NS2A, NS2B, NS3, NS4A, NS4B and NS5) [8], [9]. Following infection, the majority of neutralising antibodies are directed against the flavivirus envelope protein, although some likely recognise the pre-membrane/membrane protein [10], [11]. Antibody protection *in vivo* generally correlates with neutralising activity *in vitro* [12], [13]. In addition, cross-linking of the inhibitory receptor for the Fc portion of immunoglobulin G (IgG), FcγRIIB, can prevent antibody-dependent worsening of DENV infection, which suggests that, as long as an antibody can bind to an epitope in a manner that allows the formation of concentration-dependent viral aggregates, DENV can be neutralised *in vivo* [14]. The natural course of human infection with DENV is characterised by a broad spectrum of clinical manifestations, which includes since an asymptomatic form to mild debilitating febrile disease, known as dengue fever (DF). Shortly after defervescence, some patients develop a life-threatening syndrome characterised by spontaneous bleeding and vascular leakage that can progress to hypovolemic shock and death [15]. The risk of developing the most severe forms is related to the cross-reactive nature of the immune responses to infection with heterologous serotypes of dengue [16]. The antibody-dependent enhancement (ADE) hypothesis postulates that the formation of immune complexes among non- or sub-neutralising heterologous antibodies of DENV can worsen infection through interaction of the Fc portion of the antibody and FcγR on the surface of immune cells, such as dendritic cells, monocytes and macrophages [17]. In immune mice, Yamanaka *et al* showed that monoclonal antibodies (mAbs) against the envelope protein of DENV-2 or DENV-4 show enhancing activities at sub-neutralising doses under normal ADE assay conditions [18]. The authors found that the inclusion of commercial rabbit complement or fresh sera from healthy humans reduced enhancing activities, thereby decreasing the percentage of infected cells. The role of the complement was confirmed by addition of heat-inactivated human serum or commercial C1q- or C3-depleted human sera, which decreased enhancing activities, thereby increasing the percentage of infected cells. These results indicate that a single antibody species plays two distinct roles (neutralising and enhancing), depending on the level of complement [18].

The complement system consists of a tightly regulated network of more than 30 circulating and membrane-bound proteins, whose activation leads to the coating of foreign antigens with the key complement fragments, C3b and, to a lesser degree, C4b [19]. It is well understood that the complement system plays a role in protection against pathogens by activation of its proteolytic cascade, which results in the adherence of opsonins (C1q, C3b, iC3b and C3d[g]) to the surface of microbes; the release of pro-inflammatory peptides (the anaphylatoxins C3a and C5a); and the assembly of the membrane attack complex (terminal complement complex, or C5b-9). These activation products are the ligands of various complement receptors (CRs), namely C3aR, C5aR, CR1 (CD35), CR2 (CD21), CR3 (CD11b), CR4 (CD11c) and CRIg, which are expressed by a wide variety of immune cells [20]. Their activation occurs by one of three pathways: classical, lectin or alternative. The classical pathway is activated by interaction of C1q with the Fc portion of the IgM or IgG immune complex, although activation can also be achieved in an antibody-independent manner by some membrane components of viruses, bacteria and fungi. The lectin pathway is first activated when mannose-binding lectin, a structural homologue of C1q, binds to carbohydrate moieties on the surface of pathogens, including yeast, bacteria and viruses. The alternative pathway is spontaneously activated on biological surfaces, as well as in plasma and other body fluids, when the level of C3 hydrolysis remains consistently low. This spontaneous cleavage readily initiates amplification of the activation cascades [19]. In addition, recent studies have shown that the complement system is a key factor in coordinating important events during the immune response [21], facilitating antigen identification and localisation; cell activation, proliferation and apoptosis; B-cell triggering; and immunological memory as a bridge between innate and adaptive immunity.

A variety of aspects related to the activation and regulation of the complement system during dengue infection remain unknown. Nevertheless, like other viruses, DENV has developed mechanisms to evade the complement system and establish itself. Within this context, a recombinant soluble NS1 produced from DENV-infected cells has been shown to interact with the human complement inhibitory factor clusterin, which naturally inhibits the formation of the terminal complement complex [22]. In addition, flavivirus NS1 has been shown to play a role in limiting complement activation by forming a complex with C1s and C4 to promote cleavage of C4 to C4b or even by direct association with C4b binding protein (C4BP), a complement-regulatory plasma protein that attenuates the classical and lectin pathways [23]. Taken together, these findings further define the immune evasion potential of NS1 in reducing the functional capacity of complement activation and the control of flavivirus infection. Clinical studies have shown that the levels of DENV NS1 and products of complement activation, including those with known vascular effects, such as C3a, C5a [24] and SC5b-9 [25], are present at higher levels in patients with dengue haemorrhagic fever before vascular leakage takes place, supporting the theory that complement activation is involved in the development of severe disease and unfavourable outcomes.

In this study, our initial objective was to determine the profile of CR expression on monocytes from dengue infected patients and the degree of CS activation by SC5b-9. We correlated those data with the severity and duration of disease. Our preliminary findings prompted us to evaluate the influence that the complement system has on monocytes infected *in vitro* with DENV-2.

## Materials and Methods

### Study population

Between 2010 and 2011, we evaluated 66 patients suspected of DENV infection and treated at one of two hospitals in Brazil: the Plantadores de Cana Hospital/Dengue Referral Centre, in the city of Campos dos Goytacazes, located in the state of Rio de Janeiro; and the Federal University of Mato Grosso do Sul Professora Esterina Corsini Day Hospital, in the city of Campo Grande, located in the state of Mato Grosso do Sul. Samples were collected during the acute phase of infection (1–11 days after symptom onset). Cases of dengue were classified, according to World Health Organization criteria established in 2009, as DF (*n* = 34); DF with warning signs (DF/WS, *n* = 17); or severe DF (*n* = 15). The DF category included cases in which the diagnosis had been confirmed through laboratory tests and the patient presented with symptoms such as fever, headache, retro-orbital pain, myalgia, arthralgia, rash, nausea and vomiting, as well as being tourniquet test positive, showing leucopenia and not presenting with any warning signs. In addition to these symptoms, the patients whose cases were included in the DF/WS category had abdominal pain or tenderness; persistent vomiting; clinically relevant fluid accumulation; mucosal bleeding; lethargy; restlessness; liver enlargement (>2 cm); and an increase in haematocrit concomitant with a rapid decrease in platelet count. The severe DF category included cases characterised by severe vascular leakage leading to shock; fluid accumulation with respiratory distress; severe haemorrhage, such as like massive vaginal bleeding (in women of childbearing age); gastrointestinal bleeding; and organ impairment, such as liver damage (aspartate aminotransferase or alanine aminotransferase >1000 U/L); central nervous system involvement (drop in the level of consciousness); and the involvement of other organs, such as the heart [26]. One of the patients with severe DF developed shock and evolved to death. Demographical, clinical and biochemical data related to the patients are shown in Table 1.

**Table 1:** Demographic characteristics of the study population and clinical course of the patients with dengue.

Characteristic	Controls	Patients		
		DF	DF/WS	Severe DF
	(*n* = 10)	(*n* = 34)	(*n* = 17)	(*n* = 15)
Gender, F:M	8∶2	17∶17	10∶7	7∶8
Age (years), mean ± SD	38.4±16.5	39.8±17.5	40.4±17.1	40.4±19.6
Post-infection day^a^, mean ± SD	-	3.8±2.2	3.9±2.8	4.5±2.2
Primary infection^b^, %	-	20.6	0	0
Secondary infection^b^, %	-	79.4	100	100
Clinical sign and symptoms				
Bleeding, %^c^	-	29	53	73.3
Fluid leakage, %^d^	-	0	0	53.3
Hypotension, %^e^	-	0	0	20
Laboratory test results				
Platelets ×10^3^/mm^3^, mean ± SD	295.2±38.7	138.9±76.8*	73.9±52.5* ^,^ †	47.1±61.3* ^,^ † ^,^ ‡
Haematocrit, %, mean ± SD	37.8±1.3	40.2±3.4	38.9±5.1	36.6±3.2* ^,^ †
Total leukocytes/mm^3^, mean ± SD	6100±731.4	4047.8±2573.9*	3402.7±1426.6*	4598.5±2986*
Monocytes/mm^3^, mean ± SD	432.7±99.8	454.9±375.5	252.4±156.0	241.4±185.6
AST, IU/L, mean ± SD	nd	77.6±81.4	98.5±93.9	179±239†
ALT, IU/L, mean ± SD	nd	71.8±76.6	83.3±75	190±286†

DF, dengue fever; DF/WS, DF with warning signs; AST, aspartate aminotransferase; ALT, alanine aminotransferase; nd, not determined.

a Days from symptom onset until the interview;

b primary infection was defined as IgM antibody level >1.0 and IgG/IgM antibody ratio <0.5. Secondary infection was defined as IgG antibody level >1.0 and IgG/IgM antibody ratio >1.0 or IgM antibody level <1.0 and IgG antibody level >1.0.

c includes skin haemorrhages, epistaxis, gingival bleeding, gastrointestinal bleeding, urinary tract haemorrhage or metrorrhagia;

d signs of vascular leakage (pleural or pericardial effusion, ascites);

e pulse pressure <20 mmHg or hypotensive for age.

*p≤0.05 vs. Controls;

† p≤0.05 vs. DF;

‡ p≤0.05 vs. DF/WS. Statistical differences were assessed by Mann-Whitney U test.

We included only patients in whom a diagnosis of infection with DENV was confirmed by serological and virological methods: detection of anti-dengue IgM or IgG with capture enzyme-linked immunosorbent assay (ELISA; PanBio, Brisbane, Australia); detection of viral protein using the Platelia Dengue NS1 antigen enzyme immunoassay (Bio-Rad, Hercules, CA, USA); viral isolation in C6/36 cells of the mosquito *Aedes albopictus*; and detection of viral RNA by reverse-transcriptase polymerase chain reaction. We also enrolled 10 healthy individuals who had had no episodes of fever in the last three months and had no history of other diseases.

### Ethics Statement

The study was approved by the Ethics Committee from the Instituto de Pesquisas Clinicas Evandro Chagas, FIOCRUZ (CAAE 3723.0.000.009-08). All participating subjects gave written informed consent.

### Isolation and cryopreservation of human peripheral blood mononuclear cells and plasma samples

Peripheral blood mononuclear cells (PBMCs) were obtained from 20-ml samples of venous blood collected in citrate as anticoagulant, which was used according to assay requirements, from healthy individuals and dengue-infected patients. The blood was mixed with Ficoll-Hypaque density gradient (Sigma-Aldrich, St. Louis, MO, USA), at 1,077 g/ml. After centrifugation at 400 g for 30 min, plasma samples were skimmed from the top of the solution and PBMCs were recovered from the underlying layer. The PBMCs were washed twice in RPMI 1640 medium. After trypan blue exclusion, PBMC viability was greater than 95%. To approximately 10^6^ cells, we added freezing solution containing 90% inactivated foetal calf serum (Gibco; Life Technologies, Gaithersburg, MD, USA) and 10% dimethyl sulphoxide (Sigma Chemical Co., St. Louis, MO, USA). The plasma samples were stored at −70°C until use, and the PBMCs were stored for 24 h at −70°C, after which they were cryopreserved in liquid nitrogen for subsequent study.

### Antibodies and reagents

The mouse anti-human mAbs to be used in the flow cytometry studies were purchased as follows: CD14-phycoerythrin-cyanine (PE-Cy)5.5 (clone 61D3; Southern Biotech, Birmingham, AL, USA)—CR1 (CD35)-PE (clone E11; Serotec, Oxford, UK) or CR1 (CD35)-fluorescein isothiocyanate (FITC, clone E11; Biolegend, San Diego, CA, USA)—CR2 (CD21)-allophycocyanin (APC, clone HB5; eBioscience, San Diego, CA, USA)—CR3 (CD11b)-PE-Cy7 (clone ICRF44; Biolegend)—CR4 (CD11c)-PE (clone BU15; IOTest; Beckman Coulter, Miami, FL, USA) or CR4 (CD11c)-PE-Cy7 (clone 3.9; Biolegend)—CD59-FITC (clone MEN43; eBioscience)—CD40-APC-Cy7 (clone 5C3; Biolegend)—CD86-PE (clone 233; BD Biosciences PharMingen, San Diego, CA USA)—human leukocyte antigen-D region-peridinin chlorophyll A protein (HLA-DR-PerCp, clone L20; R&D Systems, Minneapolis, MN, USA). We employed and anti-DENV complex (Millipore, Billerica, MA, USA) labelled with Alexa-Fluor647 or Alexa-Fluor488 Zenon IgG labelling kits (Molecular Probes, Eugene, OR, USA) and FAM FLICA Caspase 1 Assay Kit (ImmunoChemistry Technologies, Bloomington, MN, USA). The isotype-control mAbs were purchased from either Biolegend or BD Biosciences Pharmingen. The following antibodies were used in the blocking assay: anti-CR1 (CD35, clone J3D3; Beckman Coulter)—anti-CR3 (CD11b, clone ICRF44; Biolegend)—anti-CR4 (CD11c, clone 3.9; Biolegend)—anti-CD18 (clone TS1/18; Biolegend)—and IgG isotype control (clone MOPC-21; Biolegend).

### Isolation and cryopreservation of human peripheral blood mononuclear cells and plasma samples

Peripheral blood mononuclear cells (PBMCs) were obtained from 20-ml samples of venous blood collected in citrate as anticoagulant, which was used according to assay requirements, from healthy individuals and dengue-infected patients. The blood was mixed with Ficoll-Hypaque density gradient (density 1,077 g/ml Sigma-Aldrich, St. Louis, MO, USA). After centrifugation at 400 g for 30 min, plasma samples were skimmed from the top of the solution and PBMCs were recovered from the underlying layer. The PBMCs were washed twice in RPMI 1640 medium. After trypan blue exclusion, PBMC viability was greater than 95%. To approximately 10^6^ cells, we added freezing solution containing 90% inactivated foetal calf serum (Gibco; Life Technologies, Gaithersburg, MD, USA) and 10% dimethyl sulphoxide (Sigma Chemical Co., St. Louis, MO, USA). The plasma samples were stored at −70°C until use, and the PBMCs were stored for 24 h at −70°C, after which they were cryopreserved in liquid nitrogen for subsequent study.

### Preparation of DENV-2 stock and titration in C6/36 cell line cultures

Preparation of ultracentrifuged DENV-2 (Thai strain 16681), kindly provided by Dr, S. B. Halstead (Naval Medical Research Center, Silver Spring, MD, USA), and determination of viral titres have been described previously [27]. The ultracentrifuged DENV-2 was concentrated 20-fold (from the initial supernatant volume) in RPMI with 10% FBS, filtered through a 0.22-µm pore-size membrane and stored at −80°C. Viral titres were quantified by determining the 50% tissue culture infectious dose per ml by the Reed-Muench method using the *Aedes albopictus* (C6/36) cell line [28].

### DENV-2 infection of monocytes and blocking assay

Monocytes were cultured in 96-well microtitration plate overnight. Cells (2×10^5^/0.2 ml) were pre-incubated with CR1 (CD35), CR3 (CD11b), CR4 (CD11c), CD18 or IgG isotype control-blocking antibodies (10 µg/ml/well) for 1 h, followed by incubation with DENV-2 diluted to 10-fold, for 2 h at 37°C. Cells were then washed with serum-free RPMI and incubated for 48 h in complete medium (RPMI with 10% FBS). Prior to incubation, supernatants were harvested to determine cytokine content by ELISA or cytometric bead array assay. Alternatively, cells were recovered for flow cytometry staining.

### Flow cytometry

The expression of cell surface markers was determined in carefully thawed cryopreserved PBMCs collected from patients and controls. In brief, PBMCs were harvested, blocked for 30 min at 4°C with 1% (w/v) bovine serum albumin (Sigma-Aldrich), 0.1% (w/v) NaN_3_ (Sigma-Aldrich) and 5% inactivated human plasma in phosphate-buffered saline (PBS), pH 7.2, and re-suspended in PBS, containing 1% bovine serum albumin and 0.1% NaN_3_. The PBMCs were then incubated with fluorochrome-conjugated antigen-specific mAbs at 4°C for 30 min, washed twice and fixed in 2% paraformaldehyde solution for 20 min at room temperature. To measure the expression of intracellular molecules, cells were fixed in 2% paraformaldehyde and permeabilised by adding 0.1% saponin, after which they were incubated with fluorochrome-conjugated antibodies for 60 min. Cells were then washed twice and fixed with 2% paraformaldehyde and immersed in PBS at 4°C. Fluorescence of at least 10,000 cells per gate was measured using the flow cytometers CyAn (Dako Cytomation, Fort Collins, CO, USA) or FACsAria (BD Biosciences PharMingen). The analysis was performed using Flow Jo software, version 7.6.1 (TreeStar, Inc., Ashland, OR, USA).

### ELISA

In plasma samples from DENV-infected patients and healthy controls, we used ELISA in order to quantify SC5b-9 (Quidel, Santa Clara, CA, USA), in accordance with the manufacturer instructions. In the supernatants of monocytes cultured under the various conditions described, we also used ELISA in order to determine the levels of DENV NS1 antigen (Platelia Dengue NS1 AG; Bio-Rad) and IFN-alpha (IFN-α; PBL InterferonSource, Piscataway, NJ, USA), in accordance with the manufacturer instructions.

### Cytometric bead array

We quantified interleukin (IL)-2, IL-4, IL-5, IL-10, TNF-α and IFN-γ in monocyte culture supernatants using the human T helper 1/T helper 2 (Th1/Th2) cytokine cytometric bead array kit (BD Biosciences PharMingen), in accordance with the manufacturer instructions, and the FACSCalibur flow cytometer (BD Biosciences PharMingen). The individual standard curve range for a given cytokine defines the minimum and maximum quantifiable levels using the kit as 20 pg/ml and 5,000 pg/ml, respectively.

### Statistical analysis

Statistical analyses were performed using GraphPad prism software, version 5.0 (GraphPad Software Inc., San Diego, CA, USA). The Mann-Whitney *U*-test was used. Correlation was estimated by Spearman rank correlation. Values of p<0.050 was taken as being significant for all statistical analyses.

## Results

### Patient characteristics

Demographical, clinical and biochemical data related to the patients enrolled in this study are shown in Table 1. Age and duration of disease at admission were comparable among the three categories (DF, DF/WS and severe DF). The majority of cases (77.8%) were DENV-2 infections, with secondary infection. Among the severe DF cases, we documented serious manifestations, such as bleeding, vascular leakage and hypotension, not seen in the other cases studied. It is of note that the total leukocyte counts were significantly lower among the patients than among the controls. Although monocyte counts tended to decline with increasing severity of infection, the differences among the categories were not significant. Platelet counts were also significantly lower among the patients than among the controls. The patients with severe DF showed platelet counts that were lower than those observed for the DF/WS and DF patients; platelet counts were also lower in the DF/WS patients than in the DF patients, as well as being lower than the control values during the acute phase of infection. Liver enzyme levels were significantly higher (by several fold) in the severe DF cases than in the DF cases.

### CRs are down-regulated on circulating monocytes in DENV infection

**Figure 1:**
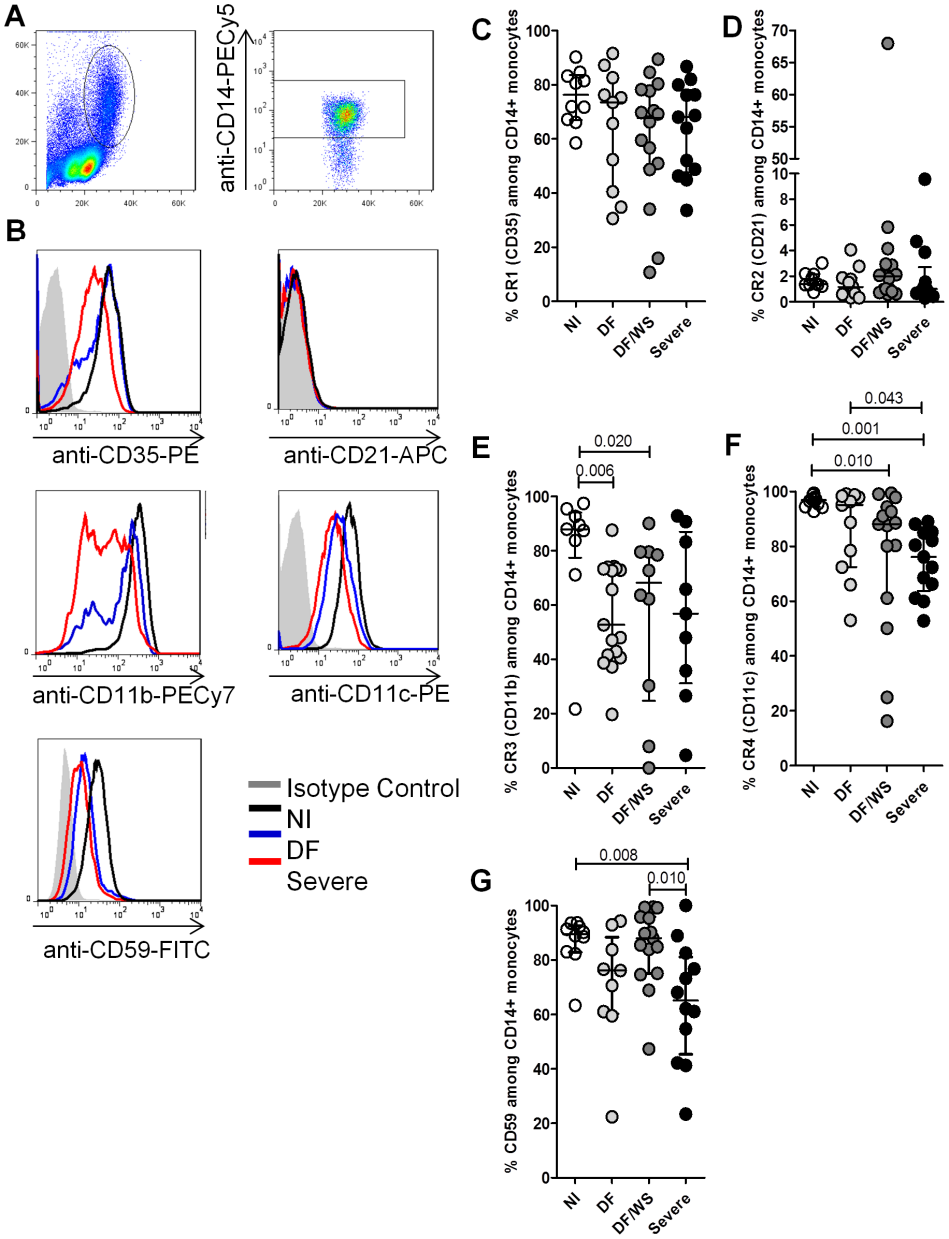
Expression of complement receptors on monocytes from DENV-infected patients and healthy controls. Flow cytometry procedures were performed to determine complement receptor (CR) expression on monocytes from patients compared with those obtained from non-infected (NI) individuals (controls). (A) The analysis of monocytes was performed by establishing a specific scatter gate using the combination of anti-cell surface antigens and laser forward scatter (FSC) to discriminate, and the monocytes were gated as FSC High (300–500) CD14^High+^. (B) The expression of CR markers was performed using the percentage determined by histogram distribution applied on events *versus* FL1/FITC (CR1 [CD35) and CD59), FL2/PE (CR4 [CD11c]), FL5/PECy7 (CR3 [CD11b]) or FL8/APC (CR2 [CD21]). Representative histograms of CR+ on monocytes from a healthy control (black line), DF patient (blue line), severe DF patient (red line) and isotype control (gray line). (C–G) Graphic representations of the frequency of CR1 (CD35), CR2 (CD21), CR3 (CD11b), CR4 (CD11c) and CD59 among CD14+ monocytes population from controls (NI = O) and patients with distinct clinical forms of dengue disease (DF; DF/WS; Severe DF). Each point represents an individual analysed. The horizontal line represents the medians, and the vertical bars represent the interquartile ranges for the various populations. The Mann-Whitney U-test was used in order to analyse differences between control and patient groups. Statistically significant p-values for differences between patients and controls are shown above the pairs. SSC, side scatter.

Among the CRs evaluated, the monocyte expression of CR3 (CD11b) (p = 0.006 DF and p = 0.02 DF/WS *vs* NI), CR4 (CD11c) (p = 0.010 DF/WS and p = 0.001 Severe *vs* NI) and CD59 (p = 0.008 Severe *vs* NI) was significantly lower in the patients than in the controls. Although the monocyte expression of CR1 (CD35) and CR2 (CD21) was also higher in patients than in controls, the differences were not significant. (Fig. 1A–G). Additional comparisons among patients, by clinical severity, revealed that the expression of CR4 (CD11c) differentiate between DF and severe DF (p = 0.040). Moreover, expression of CD59 differentiate between DF/WS and severe DF (p = 0.010) (Fig. 1A–G). We found no significant differences among the categories of disease duration (1–3 days, 4–6 days and 7–11 days), in terms of the frequency of monocytes expressing CRs (Table S1).

### Predictive value of SC5b-9 for the development of severe disease during the acute phase of dengue infection

As anticipated, the median SC5b-9 level (in ng/ml) was significantly higher in plasma samples from the patients with severe DF than in those from the patients with DF (n = 15 and 25, respectively, p = 0.010), as well as being significantly higher in the DF/WS patients than in the DF patients (n = 20 and 25, respectively, p = 0.020) (Table 2). We found no significant differences in SC5b-9 levels among the categories of disease duration (Table S1). We then subdivided the DENV-infected patients into those who showed haemorrhagic manifestations or vascular leakage and those who did not. As shown in Table 3, the levels of SC5b-9 were significantly higher among the patients with haemorrhagic manifestations than among those without (n = 30 and 26, respectively, p = 0.040), as well as being higher among those with vascular leakage than among those without (n = 7 and 43, respectively, p = 0.020).

**Table 2:** Plasma levels of the complement activation product SC5b-9 in DENV-infected patients.

Groups	SC5b-9 ng/mL	p-value*
**Controls**	128 (78.2–422.3)	
**DF**	207.5 (143.5–316.7)	
**DF/WS**	319.1 (234.8–482.5)	0.020@
**Severe DF**	389.1 (303.7–472.2)	0.040# 0.010&

*Mann–Whitney U-test.

@ DF versus DF/WS;

# DENV-patient *versus* Controls;

& Severe DF versus DF.

**Table 3:** Plasma levels of SC5b-9 among patients with dengue, by clinical manifestation.

Clinical manifestation	SC5b-9 (ng/ml)	P value*
Haemorrhage (*n* = 30)		
Yes, median (range)	328.6 (243.7–434.5)	
No, median (range)	199.8 (145.5–412.2)	0.040
Vascular leakage (*n* = 26)		
Yes, median (range)	453.2 (354.7–673.9)	
No, median (range)	266.9 (174.3–403.3)	0.020

*Mann–Whitney U-test.

### Effects of plasma cytokines on monocyte expression of CRs

We found a positive relationship between TNF-α levels and monocyte expression of CD59 (r = 0.358, p = 0.03) or CR4 (CD11c) expression among monocytes (r = 0.348, p = 0.030). No such relationship was found between IFN levels and the monocyte expression of any of the CRs evaluated. It is likely that the *in vivo* effect of down-regulation of CR expression on monocytes was attributable to cytokines, such as TNF-α, and other inflammatory mediators released during infection rather than directly to DENV.

### Monocytes expressing CR3 (CD11b), CR4 (CD11c) or CD59 appear to be more susceptible to DENV infection *in vitro*

In the *in vitro* experiments, we were not able to confirm down-regulation of CR expression on DENV-infected monocytes. In fact, the median expression of CRs was comparable between non-infected and DENV-infected monocytes (*n* = 8): for CR1 (CD35) 29.1% (11.6–37.4%) vs. 12.0% (3.1–15.8%); for CR3 (CD11b) 69.6% (67.9–76.5%) vs. 69.6% (67.4–75.0%); for CR4 (CD11c) 89.1% (62.0–92.3%) vs. 89.1% (78.9–93.5%); and for CD59 83.8% (77.5–86.5%) vs. 83.0% (74.0–89.2%). For all of the CRs evaluated, the *in vitro* and *ex vivo* models were comparable, in terms of the monocyte expression observed, with the exception of CR1 (CD35), the median *in vitro* expression of which was 12.0% (3.1–15.8%), lower than the 51.7% (41.1–70.0%) observed for monocytes collected from the DENV-infected patients.

**Figure 2:**
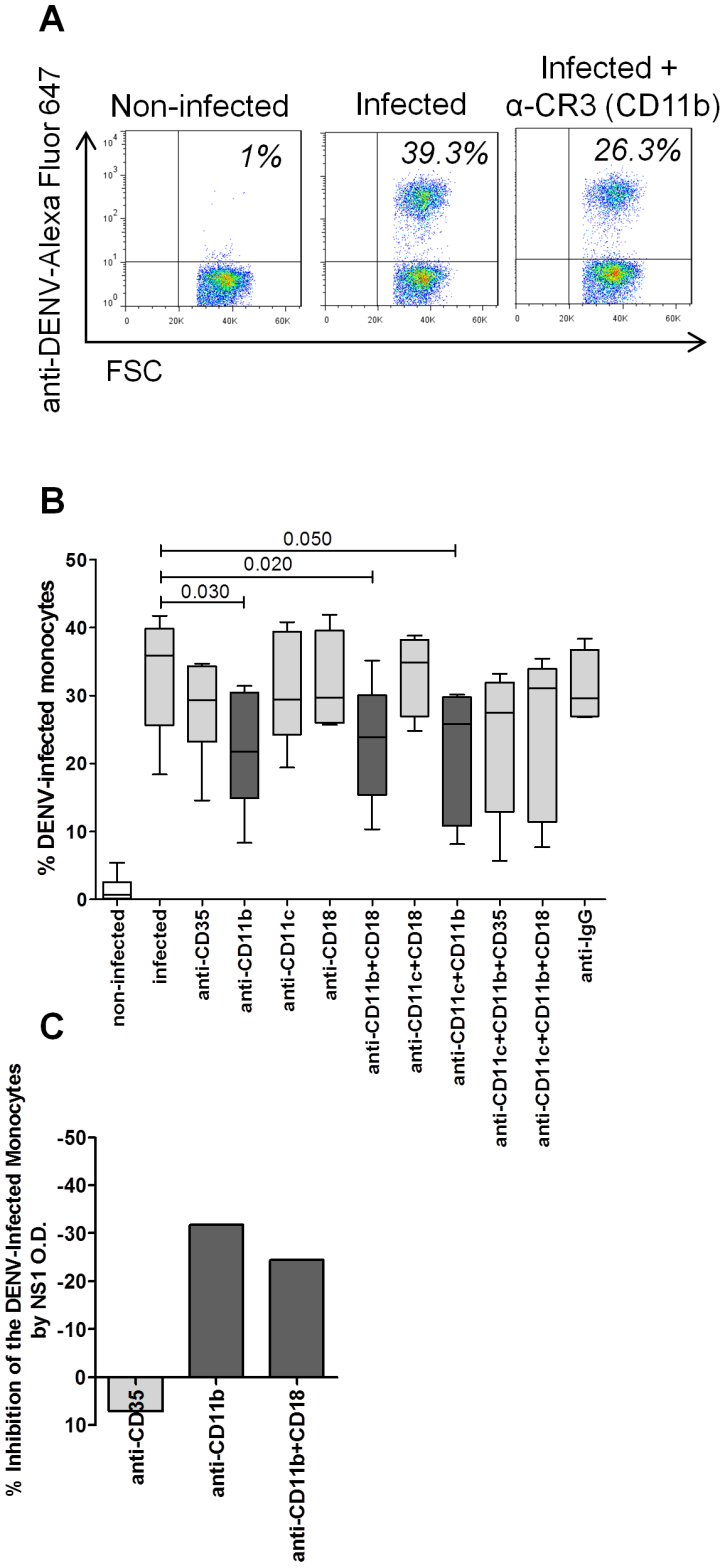
Effect of CR blocking antibody on the maturation of monocytes infected *in vitro* with DENV-2. Flow cytometry was performed to determine the frequency of anti-DENV complex staining on monocytes. In parallel, DENV NS1 antigen was made in the supernatant of these cultures of monocytes by ELISA. Both methods were used when monocytes were pre-treated or not with 10 µg/ml of blocking mAbs against CR1 (CD35), CR3 (CD11b), CR4 (CD11c), CD18 or IgG, alone or combined, followed by DENV-2 infection. (A) Specific gating strategies were used to select the monocyte population as Figure 1. The percentage of anti-DENV complex monocytes was determined using quadrant statistics applied on forward scatter (FSC) versus FL-8/anti-DENV complex/Alexa-Fluor647 dot-plot distributions. Representative dot plots are shown for non-infected and DENV-2-infected monocytes with or without CR3 (CD11b) blocking. (B) Box represents independent experiments in nine different subjects. Vertical bars indicate the median and interquartile range for each condition. (C) The mean percentage of DENV infection inhibition was determined by the reduction of the viral protein NS1 amounts on supernatants by the following formula: O.D. viral protein NS1 amounts condition test ×100/O.D. viral protein NS1 in DENV-2-infected monocytes reduced of the 100, utilising samples collected from 8 different subjects. OD, optical density. The Mann-Whitney U-test was used in order to analyse differences among different conditions. Statistically significant p-values for differences between patients and controls are shown above the pairs.

As can be seen in Figure 2A, primary monocytes were more susceptible than were non-infected monocytes to *in vitro* infection with DENV-2 35.9% (25.7–39.8%) vs. 0.7% (0.2–2.6%). Table 4 shows that DENV infection was highest for the monocytes expressing CR3 (CD11b) (p = 0.010), CR4 (CD11c) (p = 0.004) or CD59 (p = 0.002), whereas it was lowest for those are the expressing CR1 (CD35) (p = 0.002).

**Table 4:** Infection of monocytes with the dengue virus *in vitro*, by monocyte expression of the various complement receptors.

Monocyte expression	% monocytes infected	p-value* *^n = 8^*
**CR1 (CD35)**		
Positive, median (range)	5.0 (1.3–6.3)	
Negative, median (range)	29.4 (19.9–35.3)	0.002
**CR3 (CD11b)**		
Positive, median (range)	20.0 (16.7–24.7)	
Negative, median (range)	9.9 (2.2–14.3)	0.010
**CR4 (CD11c)**		
Positive, median (range)	24.2 (14.8–33.6)	
Negative, median (range)	3.7 (1.5–7.1)	0.004
**CD59**		
Positive, median (range)	24.8 (19.8–37.5)	
Negative, median (range)	3.5 (2.1–5.1)	0.002

*Mann–Whitney U-test.

### *In vitro* blocking of CR3 (CD11b) decreases DENV infection in monocytes

We found that DENV-2 infection was not significantly affected by the blocking of CR1 (CD35)- 29.3% (23.2–34.4), CR4 (CD11c)- 29.4% (24.3–39.4), CD18- 29.7% (26.0–39.6) or CR4 (CD11c/CD18)- 34.9% (27.0–38.2) with not significant inhibition of DENV-2 infection (35.9% (25.7–39.8)). In contrast, the blocking of CR3 (CD11b)- 21.8% (14.9–30.5) or CR3 (CD11b/CD18)- 23,9% (15.4–30.1) suppressed DENV-2 infection (p = 0.030 and p = 0.020, respectively). Isotype-matched antibody controls had no effect on infection (Fig. 2A,B).

We confirmed that the CR3 (CD11b) blocking antibody alone suppressed up to 32% of DENV-2 infection, as measured by the determination of NS1 production in culture supernatants on post-infection day 2 (Figure 2 C).

### Blocking of CR3 (CD11b) and CR1 (CD35) has minimal effects on the maturation of monocytes during DENV infection

We found no statistical significant effect when we compared non-infected and infected monocytes in terms of the expression of CD86 (38.6% [19.3–82.6%] vs. 69.4% [45.4–80.1%]) and HLA-DR (84% [80.7–84.9%] vs. 86.6% [76.2–92.3%]), although the expression of CD40 was lower in non-infected monocytes than in infected monocytes (21.3% [11.9–27.2%] vs. 39.9% [33.8–41.5%], p<0.030). Administered before infection, neither anti-CR1 (CD35) nor anti-CR3 (CD11b) affected the stimulatory capacity of monocytes, as evaluated by expression of CD86, CD40 and HLA-DR (data not shown). Data summarised in Table 5 demonstrate that neither infection, nor blocking of anti-CR1 (CD35) nor blocking of anti-CR3 (CD11b) significantly altered the phenotype of monocytes. This indicates that cross-linking of CR1 (CD35) or CR3 (CD11b) does not induce the maturation of DENV-2-infected monocytes. So, CR3 (CD11b) reduced DENV infection but did not affect the presentation of major histocompatibility complex class II molecules or the expression of co-stimulatory molecules.

**Table 5:** Effect of complement receptor blocking on the presentation of major histocompatibility complex class II molecules and the expression of co-stimulatory molecules on monocytes infected with the dengue virus.

Co-expression	No blocking	Blocking with anti-CD35	Blocking with anti-CD11b
	% monocytes infected	% monocytes infected	% monocytes infected
CR1 (CD35)^+^CD86^+^, median (range)	3.2 (1.9–6.2)	2.0 (1.3–3.5)	6.3 (2.8–7.6)
CR3 (CD11b)^+^CD86^+^, median (range)	19.6 (11.4–43.0)	26.8 (18.6–59.3)	23.6 (14.4–44.3)
CR4 (CD11c)^+^CD86^+^, median (range)	13.2 (10.8–30.0)	13.4 (9.8–34.6)	13.9 (12.6–23.0)
CD59^+^HLA-DR^+^, median (range)	85.7 (67.2–93.2)	89.2 (63.9–97.3)	89.9 (66.7–95.0)
CD59^+^CD40^+^, median (range)	5.0 (4.2–17.0)	5.3 (1.4–8.9)	4.4 (3.4–9.1)

### The attenuation of infection by the CR3 (CD11b) blocking was mediated by protective mechanisms induced by the anti-viral, pro-inflammatory cytokines IFN-α and TNF-α, but not caspase-1

**Figure 3:**
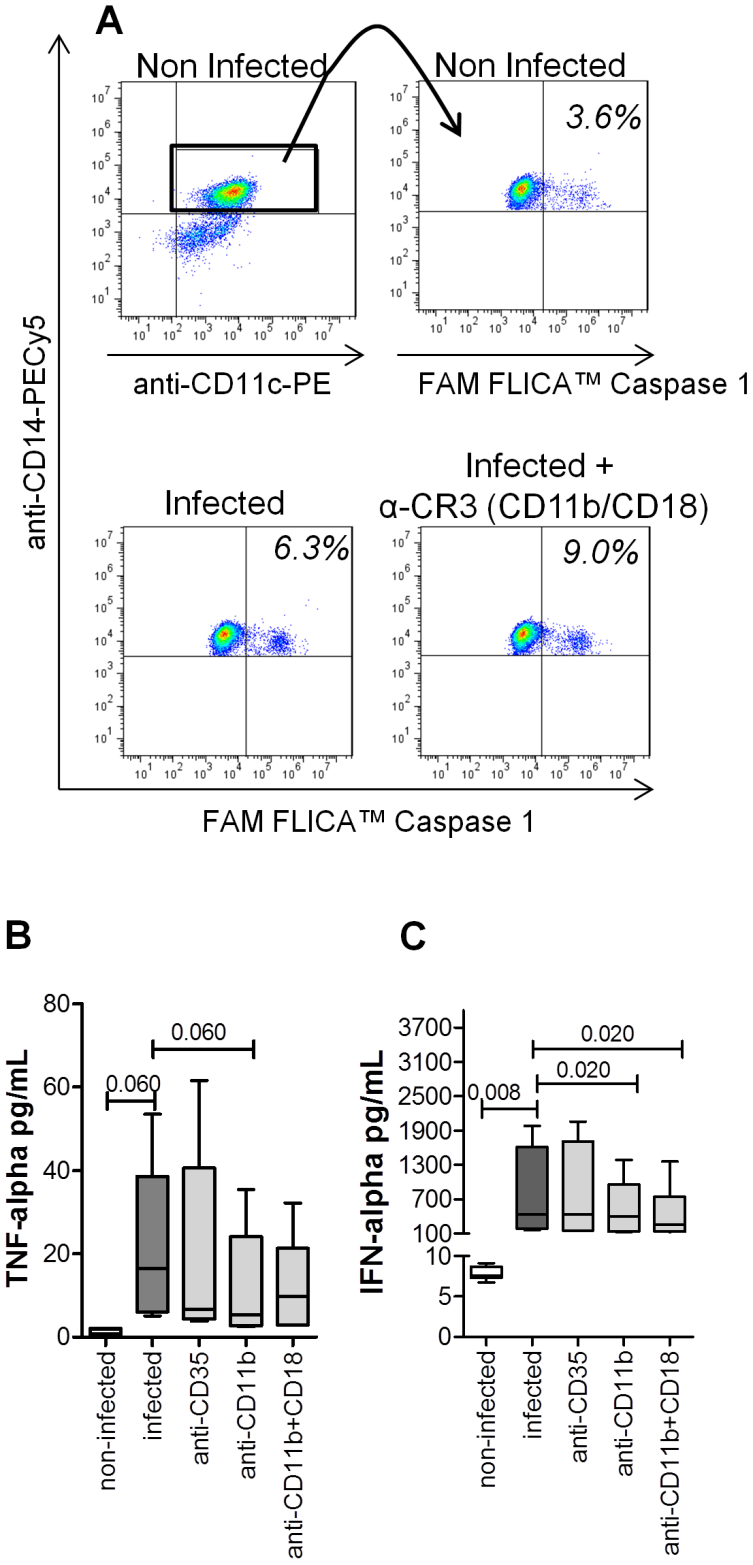
Effect of CR blocking antibody on the cytokine production and activation of caspase-1 of monocytes infected *in vitro* with DENV-2. (A) The specific scatter gate using the combination of side scatter and forward scatter, followed by the percentage using quadrant statistics applied on FL5/PECy5.5 (CD14) versus FL2/PE (CD11c) dot-plot distributions for caspase-1 analysis. Representative FAM FLICA Caspase 1 marker was quantified using the percentage determined by histogram distribution plots from analysis of the monocytes that were non-infected (NI), infected and infected but pre-treated with CR3 (CD11b/CD18) blocking antibody. Supernatant concentrations of TNF-α (B) and IFN-α (C) were quantified by CBA and ELISA, respectively, after DENV-2 infection of monocytes. The mean percentage reduction of both cytokines when cultures were pre-treated with CR blocking antibody is shown (C). The Wilcoxon-test was used in order to analyse the differences among the various conditions. Statistically significant p-values for differences between patients and controls are shown above the pairs.

We found that caspase-1 activation was greater on DENV-2-infected monocytes than on non-infected monocytes—6.1% (5.7–6.7%) vs. 3.6% (3.2–4.4%)—a difference that was significant (p<0.001). However, the blocking of CR3 (CD11b) and CR3 (CD11b/CD18) indicated a tendency to increase the levels of active caspase-1 on DENV-2-infected monocytes—7.9% (6.3–8.9%) and 8.1% (2.8–9.3%), respectively—probably ruling out inflammasome-induced production of IL-1β or IL-18 (Fig. 3A). The monocytes infected with DENV-2 did not show significant production of IL-2, IFN-γ, IL-4 or IL-5 (data not shown). However, on post-infection day 2, the production of TNF-α was significantly higher in DENV-2-infected monocytes than in non-infected monocytes—16.4 pg/ml (6.1–38.6 pg/ml) vs. 1.9 pg/ml (0.7–2.1 pg/ml) (p = 0.060)—as was that of IFN-α—432.1 pg/ml (184.3–1609.0 pg/ml) vs. 7.6 pg/ml (7.3–8.7 pg/ml) (p = 0.008)—as shown in Figure 3 (B,C). In DENV-2-infected monocytes, the blocking of CR1 (CD35), CR3 (CD11b) and CR3 (CD11b/CD18) decreased the production of TNF-α by 4.0%, 46.0% (p = 0.060) and 49.0%, respectively, and that of IFN-α by 9.5%, 26.0% (p = 0.02) and 38.0% (p = 0.020), respectively.

## Discussion

Our study revealed that the monocyte expression of CR3 (CD11b), CR4 (CD11c) and CD59 is significantly reduced in DENV-infected patients, whereas plasma levels of SC5b-9 are elevated, markedly so in patients with pronounced haemorrhagic manifestations and vascular leakage, suggests that complement activation is strongly linked to unfavourable outcomes in dengue. This allows a clear view of the relationship between disease activity and down-regulated expression of these CRs or high complement system activation by SC5b-9. The modified monocyte expression of CRs in DENV-infected patients prompted us to investigate the role of these systems in the DENV infection *in vitro* of the isolated monocytes from healthy subjects. Our data indicate that prior blocking with CR3 (CD11b) could reduce viral infection. We also found evidence that the attenuation of infection was mediated by protective mechanisms induced by the anti-viral, pro-inflammatory cytokines IFN-α and TNF-α, but not caspase-1.

Although the pathogenesis of DENV infection remains controversial, cross-reactive antibodies and effector T cells have been implicated [29], [30], [31] and it has been suggested that complement activation plays a role. Early clinical studies demonstrated that DENV-infected patients, especially those with severe DF, show reduced levels of C3, C4 and factor B, together with increased catabolic rates of C3a and C1q [32], [33]. In addition, it has been reported that C3 degradation products and anaphylatoxins accumulate in the circulation of severely ill patients and peak on the day of maximum vascular leakage [34], [35]. To our knowledge, this is the first study to address CRs expression on the surface of phagocytes in human dengue infection, although the approach taken here has been employed in studies of other diseases. One very interesting approach, albeit not applied to any specific disease, was that taken by Li *et al* [36]. Those authors demonstrated that monocyte-derived dendritic cells, under steady-state conditions, can produce a wide range of complement components, receptors and regulators, indicating that those cells are not only a significant local source of complement but are also capable of detecting soluble complement effector molecules (C3a and C5a) or their membrane-bound counterparts (C3b and its metabolite iC3b). Furthermore, the authors demonstrated that the expression of complement components, receptors and regulators is differentially regulated by lipopolysaccharide and by other TNF-α-, IL-1- or prostaglandin E_2_-related inflammatory stimuli, conditions mimicking infection and tissue inflammation [36]. No significant correlation was observed between the frequency of CR3-expressing monocytes and TNF serum levels from dengue patients, besides in vitro blockage of CR3 reduced the release of TNFα by DENV-2 infected monocytes. Recalling, the receptor for the iC3b fragment of complement, CR3, is involved in monocytes/macrophages and also with neutrophils phagocytosis. Berger and colls [37] showed that supernatants of human mononuclear cells and pre-stimulated with lipopolysaccharide or purified protein derivative contained both TNF and IL-1 and increased CR1 and CR3 expression on isolated polymorphonuclear neutrophils. Interestingly, recombinant TNF had more effect on receptor expression than IL-1, indicating that TNF is the major monocyte product that increases CR1 and CR3 expression on mature blood neutrophils. We speculate that in our cohort of dengue-infected patients, independently of CR3 expression on monocytes, monocytes remain as an important source of TNF, inducing an important systemic effect that would help the host to eradicate infection. In the other hand, CR3 can also interact with the low affinity receptor for Ig (CD16) on monocytes [38], that are the major dengue virus infected cells in the blood. Wong KL and colls. [39] found that both CD16(−) as CD16(+) monocyte subsets were equally susceptible to dengue virus. However, CD16(+) monocytes were the major producers of IL-1β, TNF-α, IL-6, CCL2, 3 and 4 in response to dengue virus. So, herein we speculate that the blocking of CR3 during in vitro infection by dengue virus indirectly acts in CD16 surface of the monocytes, interfering on its TNF production.

Therefore, complement activation itself can lead to auto-amplification or auto-regulation of the key complement components and receptors expressed in the pathogen-related and inflammatory environment, such as within the context of dengue.

The accumulation of circulating immune complexes is frequently observed in malaria. Monocytes and macrophages obtained from *Plasmodium yoelii*-infected mice have been shown to present specific inhibition of complement-mediated internalisation of immune complexes caused by the decreased CR1 (CD35) expression [40]. Similarly, the levels of surface CR1 on peripheral monocytes, macrophages and B cells from malaria patients show a significant decrease compared with uninfected control individuals from the same geographic area, suggesting that this decrease in CR1 expression plays an essential role in impaired immune complex clearance during malaria [40]. Although only small amounts of circulating immune complexes have been detected in DENV-infected patients [34], it has been hypothesised that the immune complexes composed of virions and DENV-specific antibodies activate the complement system, to deleterious effect [32]. It has also been hypothesised that NS1 or the envelope protein (the key surface viral proteins) facilitate immune complex formation and complement deposition on infected cells [25], [41]. Regarding DENV infection *per se*, FcγR expressed on the monocyte surface is the key receptor molecule for viral binding and entry in the form of DENV-antibody immune complexes. However, Sun *et al* found that binding of the immune complexes does not necessarily correspond to the input amount of antibodies [42]. Therefore, it is probable that the decreased expression of CRs on monocytes makes those cells less susceptible to DENV-antibody immune complex binding, resulting in the accumulation of circulating immune complexes or deposition of immune complexes in the peripheral tissues of DENV-infected patients. Further studies are needed in order to confirm that supposition.

The most abundant CRs present on the surface of monocytes, macrophages and dendrites are CR3 (CD11b/CD18) and CR4 (CD11c/CD18), which belong to the β_2_-integrin family. Both are able to bind C3 and C4 fragments (C3b and C4b) on a pathogen, facilitating binding and phagocytosis, a process called opsonisation, which helps clear microbial infections. In addition, these integrins mediate important functions during leucocyte extravasation across the endothelium and interaction with extracellular matrix [43], [44]. It is probable that the down-regulation of CR3 (CD11b) and CR4 (CD11c) on the monocytes of DENV-infected patients makes these cells less susceptible to activation products and therefore more susceptible to infection. Besides, these modulatory effects of CRs on monocytes can direct or indirectly influence T and B cell responses through alterations of co-stimulatory molecule signalling and cytokine expression.

The relevance of complement has received attention in studies that have indicated their critical role in vascular leakage, the major clinical complication of the pathogenesis of dengue, which occurs frequently in patients who develop the severe form of the disease. In fact, DENV-infected endothelial cells activate human complement in the presence of antibodies resulting in C5b-9 deposition [45]. In addition, NS1, the major non-structural DENV protein, has been shown to be a major trigger of complement activation, even in the absence of direct viral infection. Antibodies against NS1 direct complement attack on the infected cells, resulting in membrane damage by the C5b-9 and bystander SC5b-9 complexes. Furthermore, DENV infection can also induce the production of inflammatory cytokines [25]; membrane-associated C5b-9 might trigger cellular reactions and the production of inflammatory cytokines [46]; and SC5b-9 can independently provoke other local and systemic effects [47], [48]. Therefore, complement-activation products and cytokines might synergise locally to incur vascular leakage. Our findings are in agreement with those of Avirutnan *et al* [25], who found that DENV-infected patients show elevated plasma levels of SC5b-9, especially in patients at high risk for developing bleeding and vascular leakage. Consequently, SC5b-9 could serve as predictive marker.

The complement system is in a constant state of low-level, “tick-over” activation, which allows a rapid response to a warning sign, because any disturbance in its delicate balance can result in tissue injury. The system remains harmonious due to the influence of regulatory proteins, such as CD59, presenting in the soluble form or membrane-bound in most cells [49]. One interesting finding of the present study was that CD59 expression was decreased on monocytes from DENV-infected patients. Protectin (CD59) is expressed ubiquitously and is integrated into the cell membrane by glycosylphosphatidylinositol anchors. It regulates the formation of the terminal lytic membrane attack complex by inhibiting the interaction of the C8α-subchain and the first molecule of C9, effectively preventing integration into the cell membrane and the creation of a transmembrane pore [49], [50], [51], [52]. It is probable that, despite high plasma levels of SC5b-9, down-regulation of CD59 on monocytes from DENV-infected patients make those cells more susceptible to the membrane attack complex. Our data show that, during *in vitro* DENV infection, CD59-expressing monocytes are more often infected than are those not expressing that CR. Further studies are warranted in order to determine the consequences that the modulation of CD59, as well as that of other CD46 and CD55 membrane-bound complement regulators, has for monocytes, non-immune cells and immune cells. However, serum protein S (vitronectin) and clusterin (Sp-40,40) hinder the formation of the lytic membrane attack complex by adhesion to the lipophilic groups of C7, thereby leading to impaired anchorage in the cell membrane. In addition, although it is well known that C5b-9 can induce cell lysis, the numerous roles of sublytic amounts of C5b-9 inserted into the membrane of nucleated cells is perhaps less appreciated. Sublytic membrane attack complex can induce secretion of multimers of endothelial von Willebrand factor [53]; stimulate endothelial prothrombinase and tissue factor activity [54]; and activate platelets and fibrin deposition, resulting in a pro-thrombotic endothelial cell surface [55]. Our preliminary data suggested that high production of SC5b-9 could act on the endothelia of vessels inducing cell permeability alteration, as measured by LDH release (data not shown). Further studies are needed in order to fully explore what effect plasma/serum of DENV-infected patients has on the presence of complement blockers in endothelial cells and thus evaluate morphological changes and permeability of the endothelium.

Monocytes are natural host cells for DENV [56], [57]. Monocytes induce the production of antiviral factors, such as nitric oxide [58] and IFN-α [59], in response to infection. Nevertheless, monocytes promote dengue pathogenesis, especially during secondary immune responses, in which monocyte infection can be facilitated through ADE, leading to increases numbers of infected cells and higher viral loads [60], [61] In addition, monocytes and macrophages can produce cytokines and chemokines that compromise the integrity of the endothelial cell layer [62], [63], [64], [65], possibly leading to vascular leakage, the hallmark of severe DF [60], [66]. As enveloped viruses, the DENVs enter cells through receptor-mediated endocytosis [67], [68] and rearrange cell internal membranes to establish specific sites of replication [69], [70]. A C-type lectin mainly expressed by monocyte-derived dendrites [71], named dendritic cell-specific intercellular adhesion molecule 3-grabbing non-integrin, or CD209, is considered to be one of the most important receptors of DENVs [71], [72]. Recently, various molecules involved in the process of DENV entry into macrophages have recently been identified, such as the mannose receptor (CD206) and the C-type lectin domain family 5, member A (CLEC5A) [64]. In CR3 (CD11b)-expressing cells, antibody and complement-dependent opsonisation of West Nile virus, another flavivirus, can, paradoxically, enhance viral infection [73], [74]. Here, we showed that blocking CR3 (CD11b) rerouted expression, post-translational processing or trafficking of DENV-2, decreasing infection by up to 30%. One interesting finding of the present study was the direct correlation observed between intracellular staining of viral antigens and secreted NS1, both methods showing a 30% reduction of infection. It is known that NS1 occurs at different cellular locations—membrane-associated; in vesicular compartments; or on the surface—and as secreted lipid-rich, extracellular (non-virion) species [75]. Many authors have reported that NS1 is an essential co-factor in viral RNA replication [76], [77] and is involved in virus assembly and maturation, because its secretion profile largely mirrors that of the envelope protein and pre-membrane protein [78], [79], [80]. Additional studies are needed in order to determine at which point (i.e., in which stage of DENV replication) CR3 (CD11b) blocking decreased the infection.

Balanced signalling through CR and receptors for the Fc portion of IgG (FcR) regulates phagocytosis, degradation, and clearance of antigens/immune complexes, as well as antigen presentation, antibody-dependent cell-mediated cytotoxicity, cytokine expression, oxidative stress, and distribution or differentiation of monocytes [81], [82], [83], [84]. In the present study, we characterised the functional aspects of infected monocytes with or without blocked CRs, as well as cell activation and cytokine production, in order to further the understanding of the role that the complement system and monocytes play in the pathology of DENV infection.

Sun *et al* [42] found that the binding of DENV-antibody immune complexes to monocytes up-regulated monocyte expression of CD86 and CD40, with greater production of TNF-α, IFN-α and IL-10, corresponding to infection enhancement, when compared with free DENV.

In a recent study [85], DENV was found to trigger caspase-1 secretion of IL-1β and IL-18, as well as to induce inflammatory macrophage pyroptosis. The authors of that study also showed that DENV-induced activation of the nucleotide-binding oligomerisation domain-like receptor 3 inflammasome occurs via CLEC5A, and that blocking of CLEC5A not only suppresses caspase-1-mediated maturation of IL-1β and IL-18 but also reduces pro-IL-1β [85]. We found that the blocking of CR3 (CD11b) or CR3 (CD11b/CD18) had no effect on major histocompatibility complex presentation, although it did impair secretion TNF-α and IFN-α. In addition, the blocking of CR3 (CD11b) or CR3 (CD11b/CD18) not modulated active caspase-1 on monocytes, probably ruling out inflammasome-driven production of IL-1β or IL-18.

Taken together, our data could support the hypothesis that DENV has developed strategies to evade or exploit complement in order to establish infection in humans. Such strategies include down-regulating monocyte expression of CRs such as CR3 (CD11b) and CR4 (CD11c), which could to lead to the accumulation of circulating immune complexes or the deposition of immune complexes in the peripheral tissues, as well as preventing phagocytosis and opsonisation, thus impeding viral clearance. In addition, down-regulation of host cell expression of complement regulators such as CD59 could make those cells more susceptible to the membrane attack complex. In general, complement activity decrease enhancing activities of specific antibodies by changing its threshold activity, favouring the neutralizing activity of these antibodies and viral control. Herein, our data indicated that the blockade of signalling CR3 in monocytes reduced 30% of the viral infection. We do not know yet which precise mechanisms are involved in this viral reduction. We also do not yet know if DENV complexes an antibody with neutralizing or if sub neutralizing activities could alter this activity. These issues are part of our perspective studies. A focus on the development of a novel strategy of blocking CR3 (CD11b) or CR3 (CD11b/CD18) pathways *in vitro* could have implications for the treatment of viral infection by manipulating antiviral-driven mechanisms. Future studies should focus on determining which inflammasome-independent mechanisms are associated with the reduction in viral load caused by the down-regulated expression of complement components, as well as on correlating those mechanisms with disease severity in DENV infection.

## Supporting Information

Table S1 Frequency of complement receptor expression on monocytes and plasma levels of SC5b-9, by disease duration.
(DOCX)
