# Supplementary material for: Down-Regulation of Complement Receptors on the Surface of Host Monocyte Even as In Vitro Complement Pathway Blocking Interferes in Dengue Infection
Source: PLoS One. 2014 Jul 25;9(7):e102014. doi: 10.1371/journal.pone.0102014 (PMC4111305; doi:10.1371/journal.pone.0102014)
Supplement: Table S1 — Frequency of complement receptor expression on monocytes and plasma levels of SC5b-9, by disease duration. (DOCX) [file pone.0102014.s001.docx]

**Table S1.** Frequency of complement receptor expression on monocytes and plasma levels of SC5b-9, by disease duration.

| **Variable** | **Days post-infection** | | |
| --- | --- | --- | --- |
|  | **1–3** | **4–6** | **7–11** |
| CR1 (CD35) on CD14^+^ cells (%), median (range) | 69.7 [52.3–78.2] | 64.9 [46–77.8] | 67.4 [47–78.6] |
| CR2 (CD21) on CD14^+^ cells (%), median (range) | 1.9 [0.7–3.2] | 1.0 [0.7–2.9] | 1.0 [0.8–2.3] |
| CR3 (CD11b) on CD14^+^ cells (%), median (range) | 56.8 [34.5–73.3] | 52.7 [36.5–80.8] | 76.2 [33.4–82.8] |
| CR4 (CD11c) on CD14^+^ cells (%), median (range) | 87.8 [66.7–95.3] | 79.4 [64.6–89.7] | 83.5 [70.0–94.6] |
| CD59 on CD14^+^ cells (%), median (range) | 84.4 [61.4–95.1] | 70.6 [60.3–82.8] | 83.2 [61.7–88.5] |
| SC5b-9 (ng/ml), median (range) | 299.5 [171.4–430.9] | 354.7 [211.1–497.1] | 207.5 [139.6–312.3] |
